# The contribution of pseudouridine to stabilities and structure of RNAs

**DOI:** 10.1093/nar/gkt1330

**Published:** 2013-12-24

**Authors:** Elzbieta Kierzek, Magdalena Malgowska, Jolanta Lisowiec, Douglas H. Turner, Zofia Gdaniec, Ryszard Kierzek

**Affiliations:** ^1^Institute of Bioorganic Chemistry, Polish Academy of Sciences, 61-704 Poznan, Noskowskiego 12/14, Poland and ^2^Department of Chemistry, University of Rochester, Rochester, NY 14627, USA

## Abstract

Thermodynamic data are reported revealing that pseudouridine (Ψ) can stabilize RNA duplexes when replacing U and forming Ψ-A, Ψ-G, Ψ-U and Ψ-C pairs. Stabilization is dependent on type of base pair, position of Ψ within the RNA duplex, and type and orientation of adjacent Watson–Crick pairs. NMR spectra demonstrate that for internal Ψ-A, Ψ-G and Ψ-U pairs, the N3 imino proton is hydrogen bonded to the opposite strand nucleotide and the N1 imino proton may also be hydrogen bonded. CD spectra show that general A-helix structure is preserved, but there is some shifting of peaks and changing of intensities. Ψ has two hydrogen donors (N1 and N3 imino protons) and two hydrogen bond acceptors because the glycosidic bond is C-C rather than C-N as in uridine. This greater structural potential may allow Ψ to behave as a kind of structurally driven universal base because it can enhance stability relative to U when paired with A, G, U or C inside a double helix. These structural and thermodynamic properties may contribute to the biological functions of Ψ.

## INTRODUCTION

**Figure 1.**
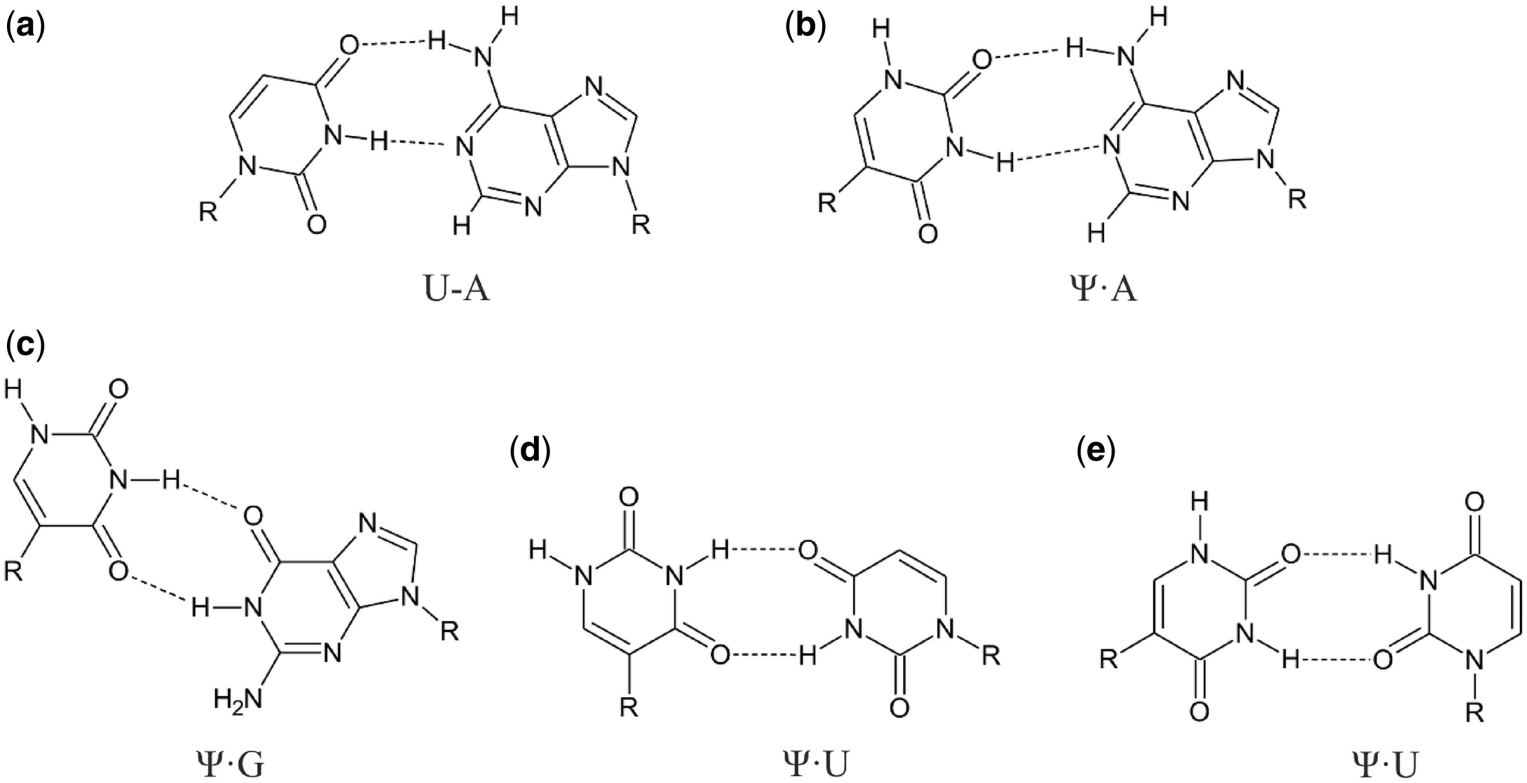
The base pairing of (**a**) uridine with adenosine and pseudouridine with (**b**) adenosine, (**c**) guanosine and (**d**, **e**) uridine.

Pseudouridine (5-ribosyluracil, Ψ) is one of the most abundant modified nucleotides (1). For example, it is found in transfer, ribosomal, small nuclear and small nucleolar RNAs (snoRNAs), and is likely important for structure and function. Isomerization of the glycosidic bond from N1 to C5 of uracil (Figure 1) enhances base rotation (1,2) and allows both N1 and N3 imino protons to serve as hydrogen bonding donors. It was postulated that Ψ could increase the thermodynamic stability of RNA duplexes due to formation of an additional hydrogen bond and/or better stacking (3,4). In comparison to uridine, Ψ favors *C3′-endo* conformation of the ribose which is correlated with *anti* conformational preferences of nucleobases (3). Published results demonstrate structural rigidity of Ψ within single- and double-stranded RNA regions, but the basic topology of U and Ψ containing RNA is usually similar (1,3–10). In a model for the U2 snRNA involved in splicing of pre-mRNA, however, Ψ-induced significant changes of the architectural landscape of the spliceosomal branch site of RNA compared with an unmodified counterpart (11,12).

Ψ is found in the TΨC loop of almost all tRNAs. For most tRNAs, Ψ is also found in the D stem and/or in the anticodon stem and loop (1). Ψ contributes to stabilization of specific structural motifs. For example, Ψ is phylogenetically conserved in major splicesomal snRNAs (U1, U2, U4, U5 and U6) and mostly located in regions involved in RNA–RNA and RNA–protein interactions essential for assembly of the splicesome and for the splicing process (12–14). The 5′ end of U2 snRNA binds directly to pre-mRNA splice sites and is particularly rich in Ψ residues (12). Structural studies of models for RNAs involved in splicing indicate that a Ψ-A pair adjacent to a splice site stabilizes the helix formed between U2 snRNA and pre-mRNA and induces bulging out of an adenosine to initiate splicing (4). The function of Ψ in snoRNAs is unknown. Ψ is also present in functionally important regions of ribosomal RNAs (small subunit, large subunit, 5.8S and 5S), including the peptidyl transferase center (PTC) (1). In eukaryotic large subunit rRNAs, Ψ accounts for 0.9–1.4% of the nucleotides (1). Positions of many Ψ in rRNAs are very conserved, suggesting importance for structure and function. For example, *Escherichia coli* rRNA helix 69 (region 1906-1924) and human rRNA region 3722-3740 contain three and five conserved Ψ residues, respectively (1,15,16). Spectral studies on a bacterial hairpin containing helix 69 show that Ψ’s exhibit a range of effects on RNA stability and structure, depending on their location (5,7,8,17). Ψ’s placed in the single-stranded loop destabilize the hairpins whereas Ψ in the helical stem increases stability.

There is limited information concerning the influence of Ψ on structure and thermodynamic stabilities of RNAs (3,5,7–9,17–19). Published data concern mostly the following topics: (i) influence of Ψ on stabilities, structure and recognition of anticodon hairpins of tRNAs (1,6,20,21); (ii) contribution of Ψ residues to structure and stability of hairpins, e.g. helix 69 and region 3722-740 of *E. coli* 23S rRNA and *Homo sapiens* 28S rRNA (5,7,8,17); (iii) importance of conserved Ψ modification in eukaryotic U2 snRNA for inducing changes in branch-site architecture of pre-mRNA at 5′-splice sites (4,9,11,14,18,22); and (iv) sequence dependence of stabilities of Ψ-A pairs (19).

Here, we present results concerning the influence of Ψ residues on thermodynamic stabilities of RNA duplexes containing Ψ-A, Ψ-G, Ψ-U and Ψ-C pairs at internal and terminal positions. The contribution of Ψ as 5′- and 3′-terminal dangling ends is also reported. Additionally, the influence of A-U and G-C adjacent pairs on the thermodynamic stabilities of RNA duplexes with a central Ψ-A or Ψ-G pair is reported. Circular dichroism (CD) spectra of Ψ containing RNA duplexes were also measured. Thermodynamic and CD results for Ψ are compared to those of isosequential duplexes with U instead of Ψ. NMR spectra of imino proton regions of RNA duplexes containing Ψ indicate that the N3 proton is usually hydrogen bonded in Ψ-A, Ψ-G and Ψ-U pairs.

## MATERIALS AND METHODS

### 

#### Choice of oligonucleotide sequences

The oligonucleotide sequences were selected to allow placing of Ψ at internal and terminal positions with the same nearest neighbor to minimize influence of surrounding Watson–Crick base pairs on stabilities, structures and interactions of Ψ pairs. Duplexes with Ψ-A, Ψ-G, Ψ-U and Ψ-C pairs were compared to otherwise identical duplexes with Ψ replaced by U.

#### Synthesis and purification of oligonucleotides

Oligonucleotides were synthesized on an Applied Biosystems DNA/RNA and MerMade12 (Bioautomation) synthesizers, using standard β-cyanoethyl phosphoramidite chemistry on solid support (23). Commercially available A, C, G, U (GenePharma) and Ψ (ChemGenes) phosphoramidites with 2′-O-tertbutyldimethylsilyl were used for synthesis of RNA. The syntheses of all oligonucleotides were performed on universal Unylinker synthesized for this purpose. The details of deprotection and purification of oligoribonucleotides were described previously (24,25). Thin-layer chromatography (TLC) purification of the oligonucleotides was carried out on Merck 60 F_254_ TLC plates with the mixture 1-propanol/aqueous ammonia/water = 55:35:10 (v/v/v). Purified oligonucleotides were characterized by mass spectrometry (MALDI TOF).

#### UV melting experiments

Oligonucleotides were melted in buffer containing 1 M NaCl, 20 mM sodium cacodylate, 0.5 mM Na_2_EDTA, pH 7. Oligonucleotide single strand concentrations were calculated from absorbance above 80°C and single strand extinction coefficients were approximated by a nearest-neighbor model (26,27). The extinction coefficient of Ψ nucleoside at 260 nm is ∼20% smaller than for U but was assumed to be equal to U (28). This results in only 1–2% error in duplex concentration. Absorbance versus temperature melting curves for nine concentrations of RNA duplexes ranging in concentration between 10^−^^3^ and 10^−^^6 ^M were measured at 260 nm with a heating rate of 1°C/min from 0 to 90°C on a Beckman DU 640 or JASCO V-650 spectrophotometer with a thermoprogrammer. Melting curves were analysed and thermodynamic parameters calculated from a two-state model with the program MeltWin 3.5, which includes standard deviation estimates of errors (29). For all sequences, except 5′UCACUGAGU/3′AGUGGCUCA, the ΔH° derived from 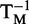 versus ln(C_T_/4) plots is within 15% of that derived from averaging the fits to individual melting curves, as expected if the two-state model is reasonable. For the exception, two transitions were observed, so the two-state approximation is not valid. Comparisons between duplexes used values from 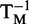 versus ln (C_T_/4) plots fit by linear least squares. Standard deviations were propagated by taking the square root of the sum of the squares of the individual standard deviations. The correlations coefficients between ΔH^o^ and ΔS^o^ were 0.99995 for duplexes with and without Ψ, as calculated from Tm's (24).

#### CD spectroscopy

CD spectra of RNA duplexes were measured in triplicate from 205 to 350 nm at 10°C and 25°C on a JASCO 815 spectropolarimeter. The buffer was 1 M NaCl, 20 mM sodium cacodylate and 0.5 mM Na_2_EDTA at pH 7 and duplex concentration was 0.12 mM. The measured CD spectra were averaged; spectrum of the buffer subtracted and the result converted into molar ellipticity per nucleotide (Δε).

#### Nuclear magnetic resonance

All RNAs used for NMR measurements were dissolved in buffer containing 150 mM NaCl, 10 mM sodium phosphate and 0.1 mM EDTA, pH 6.8. For studies of exchangeable protons, the solvent was H_2_O/D_2_O (9:1, v/v) and for assignment of aromatic protons the solvent was 99.990% D_2_O (Sigma Aldrich). All the samples were annealed by heating at 90°C for 5 min, then slowly cooled to room temperature and stored at 4°C.

NMR spectra were measured on a Bruker AVANCE III 700 MHz spectrometer, equipped with a QCI CryoProbe. The 3 mm sample tubes were used with a final sample volume of 210 µl. The 1D proton spectra in H_2_O/D_2_O (9:1, v/v) were collected with water suppression using excitation sculpting with gradients (30) and in D_2_O, with presaturation, from 128 scans. Assignment of resonances was based on homonuclear NOESY (30) and heteronuclear ^1^H-^15^N HSQC spectra (31–34). Spectra were processed and prepared with TopSpin 3.0 Bruker Software.

## RESULTS

Thermodynamic data for model RNA duplexes with Ψ and, in parenthesis, with Ψ replaced by U are presented in Table 1. The thermodynamic effects of adding terminal nucleotides were measured by extending core duplexes 5′CAGUCAGU/3′GUCAGUCA and 5′UCAGUCAG/3′AGUCAGUC (Table 2). The thermodynamic effects of replacing U with Ψ were measured in duplexes of the type 5′UCAXMYAGU/3′AGUYNXUCA, where X-Y is a Watson–Crick pair, M is U or Ψ and N is A, C, G or U (Tables 1 and 3).

**Table 1.** Thermodynamic parameters of duplex formation with pseudouridine^a^

	Average of curve fits				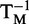 versus log C_T_ plots			
	–ΔH°	–ΔS°	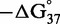	T_M_^b^	–ΔH°	–ΔS°	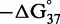 ^c^^,^^d^^,^^e^	T_M_^b^
	(kcal/mol)	(eu)	(kcal/mol)	(°C)	(kcal/mol)	(eu)	(kcal/mol)	(°C)
5′ **Ψ**CAGUCAGU 3′	78.5 ± 2.8	216.8 ± 8.5	11.23 ± 0.14	56.8	71.7 ± 1.4	195.8 ± 4.3	10.92 ± 0.06	57.2
3′ GUCAGUCA 5′	(76.3 ± 4.1)^c^	(210.2 ± 12.7)	(11.06 ± 0.21)	(56.6)	(75.8 ± 5.1)	(208.7 ± 15.8)	(11.04 ± 0.24)	(56.6)
5′ UCAGUCAG**Ψ** 3′	77.1 ± 8.3	212.7 ± 25.5	11.17 ± 0.44	56.9	72.9 ± 5.1	199.9 ± 15.8	10.93 ± 0.25	56.9
3′ AGUCAGUC 5′	(83.8 ± 12.3)	(234.5 ± 38.2)	(11.05 ± 0.51)	(54.7)	(78.7 ± 6.3)	(218.5 ± 19.6)	(10.93 ± 0.27)	(55.4)
5′ **Ψ**CAGUCAGU 3′	76.0 ± 3.4	207.3 ± 10.6	11.64 ± 0.13	59.9	84.3 ± 2.0	233.0 ± 6.1	12.07 ± 0.11 **[12.33]**	58.8
3′ **A**GUCAGUCA 5′	(92.6 ± 8.1)	(257.6 ± 24.7)	(12.68 ± 0.50)	(59.1)	(83.7 ± 5.6)	(230.7 ± 17.3)	(12.14 ± 0.34) **{12.5}**	(59.3)
5′ UCAG**Ψ**CAGU 3′	85.0 ± 7.2	233.1 ± 21.9	12.73 ± 0.37	61.4	87.2 ± 7.0	239.9 ± 21.1	12.85 ± 0.43**[13.69]**	61.2
3′ AGUC**A**GUCA 5′	(92.6 ± 8.1)	(257.6 ± 24.7)	(12.68 ± 0.50)	(59.1)	(83.7 ± 5.6)	230.7 ± 17.3)	(12.14 ± 0.34) **{12.5}**	(59.3)
5′ UCAC**Ψ**GAGU 3′	105.5 ± 8.2	293.3 ± 24.4	14.51 ± 0.32	62.4	104.9 ± 6.2	291.6 ± 18.5	14.44 ± 0.47 **[13.28]**	62.3
3′ AGUG**A**CUCA 5′	(85.4 ± 8.5)	(236.5 ± 12.1)	(12.07 ± 0.22)	(58.5)	(84.6 ± 2.8)	(234.1 ± 8.4)	(12.01 ± 0.14) **{12.5}**	(58.5)
5′ UCAA**Ψ**UAGU 3′	78.9 ± 7.3	226.8 ± 22.9	8.51 ± 0.18	45.0	71.5 ± 2.7	203.5 ± 8.7	8.36 ± 0.05 **[11.41]**	45.2
3′ AGUU**A**AUCA 5′	(83.2 ± 9.1)	(242.0 ± 28.8)	(8.16 ± 0.25)	(43.2)	(80.2 ± 8.6)	(232.5 ± 27.4)	(8.09 ± 0.27) **{7.9}**	(43.2)
5′ UCAU**Ψ**AAGU 3′	76.7 ± 8.0	220.1 ± 25.1	8.48 ± 0.19	45.1	74.1 ± 4.1	211.8 ± 13.3	8.42 ± 0.08 **[9.37]**	45.1
3′ AGUA**A**UUCA 5′	(80.1 ± 5.9)	(232.7 ± 19.2)	(7.87 ± 0.07)	(42.3)	(84.3 ± 9.2)	(246.5 ± 29.9)	(7.87 ± 0.17) **{7.9}**	(42.0)
5′ UCAGUCAG**Ψ** 3′	79.8 ± 10.2	218.7 ± 31.1	11.97 ± 0.55	59.7	82.6 ± 6.8	227.3 ± 20.9	12.06 ± 0.42 **[12.44]**	59.3
3′ AGUCAGUC**A** 5′	(92.6 ± 8.1)	(257.6 ± 24.7)	(12.68 ± 0.50)	(59.1)	(83.7 ± 5.6)	(230.7 ± 17.8)	(12.14 ± 0.34) **{12.5}**	(59.3)
5′ **Ψ**CAGUCAGU 3′	77.7 ± 4.5	213.5 ± 13.7	11.51 ± 0.28	58.2	83.5 ± 2.4	231.3 ± 7.3	11.79 ± 0.12	57.8
3′ **G**GUCAGUCA 5′	(89.4 ± 7.8)	(248.2 ± 23.7)	12.42 ± 0.46)	(58.9)	(83.3 ± 6.5)	(229.6 ± 19.7)	(12.04 ± 0.36) **{12.5}**	(59.0)
5′ UCAG**Ψ**CAGU 3′	95.8 ± 10.3	264.9 ± 30.8	13.62 ± 0.76	61.8	93.4 ± 4.2	257.9 ± 12.8	13.41 ± 0.27	61.7
3′ AGUC**G**GUCA 5′	(94.3 ± 4.2)	(263.9 ± 12.9)	(12.51 ± 0.32)	(58.0)	(84.9 ± 3.5)	(235.1 ± 10.8)	(12.01 ± 0.18) **{11.9}**	(58.4)
5′ UCAC**Ψ**GAGU 3′^f^	82.2 ± 6.6	227.2 ± 19.9	11.77 ± 0.42	58.1	79.5 ± 2.3	218.8 ± 7.3	11.61 ± 0.12	58.2
3′ AGUG**G**CUCA 5′	(105.9 ± 7.0)	(301.3 ± 20.8)	(12.49 ± 0.66)	(55.5)	(76.8 ± 1.8)	(211.5 ± 5.6)	(11.18 ± 0.08) **{11.4}**	(57.0)
5′ UCAA**Ψ**UAGU 3′	75.6 ± 5.7	218.4 ± 18.4	7.89 ± 0.14	42.7	68.4 ± 5.0	195.5 ± 15.9	7.82 ± 0.10	43.0
3′ AGUU**G**AUCA 5′	(79.5 ± 12.1)	(233.5 ± 38.6)	(7.04 ± 0.15)	(39.0)	(73.1 ± 2.4)	(213.2 ± 7.8)	(7.00 ± 0.02) **{7.3}**	(39.0)
5′ UCAU**Ψ**AAGU 3′	78.8 ± 8.5	230.2 ± 27.2	7.41 ± 0.12	40.5	70.7 ± 2.5	204.0 ± 8.2	7.40 ± 0.02	40.9
3′ AGUA**G**UUCA 5′	(71.4 ± 1.1)	(209.4 ± 3.8)	(6.41 ± 0.14)	(36.5)	(62.9 ± 3.2)	(181.6 ± 10.5)	(6.56 ± 0.07) **{6.7}**	(37.1)
5′ UCAGUCAG**Ψ** 3′	99.7 ± 5.6	277.6 ± 17.1	13.60 ± 0.35	60.7	93.1 ± 2.6	257.6 ± 7.9	13.18 ± 0.16	60.9
3′ AGUCAGUC**G** 5′	(95.9 ± 8.4)	(267.5 ± 25.5)	(12.98 ± 0.53)	(59.3)	(86.7 ± 4.2)	(239.6 ± 12.7)	(12.40 ± 0.24) **{12.9}**	(59.5)
5′ **Ψ**CAGUCAGU 3′	87.4 ± 6.8	243.4 ± 20.8	11.95 ± 0.36	57.5	82.3 ± 1.7	227.7 ± 5.2	11.67 ± 0.09	57.7
3′ **U**GUCAGUCA 5′	(78.6 ± 6.2)	(217.3 ± 19.1)	(11.24 ± 0.32)	(56.7)	(76.7 ± 4.0)	(211.5 ± 12.2)	(11.12 ± 0.18)	(56.7)
5′ UCAG**Ψ**CAGU 3′	77.2 ± 7.7	217.9 ± 24.0	9.62 ± 0.27	49.9	69.4 ± 4.2	193.7 ± 13.2	9.35 ± 0.13	50.1
3′ AGUC**U**GUCA 5′	(75.5 ± 9.4)	(214.9 ± 29.4)	(8.83 ± 0.31)	(46.8)	(65.7 ± 2.6)	(184.0 ± 8.1)	(8.58 ± 0.05)	(47.0)
5′ UCAGUCAG**Ψ** 3′	83.4 ± 5.1	231.2 ± 15.7	11.70 ± 0.27	57.5	85.7 ± 4.1	238.2 ± 12.6	11.79 ± 0.22	57.3
3′ AGUCAGUC**U** 5′	(78.7 ± 3.1)	(218.0 ± 9.5)	(11.10 ± 0.15)	(56.1)	(76.9 ± 1.2)	212.6 ± 3.6)	(11.01 ± 0.05)	(56.2)
5′ **Ψ**CAGUCAGU 3′	74.4 ± 3.8	204.1 ± 11.9	11.08 ± 0.20	57.2	74.9 ± 3.4	206.0 ± 10.6	11.07 ± 0.15	57.0
3′ **C**GUCAGUCA 5′	(78.4 ± 1.1)	(215.6 ± 3.3)	(11.56 ± 0.09)	(58.3)	(74.8 ± 1.2)	(204.5 ± 3.7)	(11.38 ± 0.06)	(58.5)
5′ UCAG**Ψ**CAGU 3′	69.6 ± 5.3	197.2 ± 17.0	8.41 ± 0.10	45.6	67.2 ± 1.5	189.8 ± 4.7	8.34 ± 0.02	45.6
3′ AGUC**C**GUCA 5′	(77.6 ± 12.3)	(223.7 ± 38.5)	(8.23 ± 0.32)	(43.9)	(69.8 ± 3.1)	(199.1 ± 9.8)	(8.07 ± 0.05)	(44.0)
5′ UCAGUCAG**Ψ** 3′	77.7 ± 3.3	214.1 ± 10.3	11.32 ± 0.16	57.4	81.6 ± 2.2	226.0 ± 6.8	11.48 ± 0.11	57.0
3′ AGUCAGUC**C** 5′	(74.9 ± 4.2)	(205.9 ± 12.9)	(11.05 ± 0.22)	(56.9)	(80.7 ± 1.4)	(223.8 ± 4.1)	(11.32 ± 0.07)	(56.6)
5′ CAGUCAGU 3′	71.0 ± 3.5	196.6 ± 11.0	10.04 ± 0.14	53.1	77.6 ± 4.0	217.1 ± 12.4	10.26 ± 0.15	52.7
3′ GUCAGUCA 5′								
5′ UCAGUCAG 3′	73.5 ± 5.0	204.5 ± 15.6	10.07 ± 0.22	52.7	71.2 ± 3.9	197.4 ± 12.3	9.96 ± 0.15	52.7
3′ AGUCAGUC 5′								

^a^Solutions are 1 M NaCl, 20 mM sodium cacodylate and 0.5 mM Na_2_EDTA, pH 7.

^b^Calculated for 10^–4 ^M total oligonucleotide strand concentration.

^c^In parenthesis the thermodynamic parameters of analogs RNA duplexes with U instead of Ψ.

^d^In square brackets the free energy of duplexes calculated according to Ψ nearest-neighbor parameters published in (19).

^e^In braces free energy of unmodified RNA duplexes calculated with RNAstructure (35,43).

^f^Two transitions were observed when central pair was U-G, so the two-state approximation is not valid.

**Table 2.** Thermodynamic increments for adding terminal nucleotides

Core duplex	Added U terminus	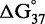 (kcal/mol)	Added Ψ terminus	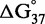 (kcal/mol)	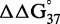 ^a^ (kcal/mol)
5′ CAGUCAGU 3′ 3′ GUCAGUCA 5′	5′U	–0.78 ± 0.28 (–0.1)^b^	5′Ψ	–0.66 ± 0.16	+0.12
	5′U/3′A	–1.88 ± 0.37 (–1.9)^c^	5′Ψ/3′A	–1.81 ± 0.17 (–2.18)^d^	+0.07 (0.2)
	5′U/3′G	–1.78 ± 0.39 (–1.8)^e^	5′Ψ/3′G	–1.53 ± 0.19	+0.25
	5′U/3′U	–0.86 ± 0.23 (–1.0)^f^	5′Ψ/3′U	–1.41 ± 0.17	–0.55
	5′U/3′C	–1.12 ± 0.16 (–0.9)^g^	5′Ψ/3′C	–0.81 ± 0.21	+0.31
5′ UCAGUCAG 3′ 3′ AGUCAGUC 5′	3′U	–0.97 ± 0.31 (–0.6)^h^	3′Ψ	–0.97 ± 0.29	0.00
	3′U/5′A	–2.18 ± 0.37 (–1.79)^c^	3′Ψ/5′A	–2.10 ± 0.45 (–2.98)^d^	+0.08 (0.1)
	3′U/5′G	–2.44 ± 0.28 (–2.15)^e^	3′Ψ/5′G	–3.22 ± 0.22	–0.78
	3′U/5′U	–1.05 ± 0.16 (1.0)^f^	3′Ψ/5′U	–1.83 ± 0.23	–0.78
	3′U/5′C	–1.36 ± 0.17 (–1.2)^f^	3′Ψ/5′C	–1.52 ± 0.19	–0.16

Thermodynamic values used are from Tm^–1^ plots.

^a^Differences between free energy increments from replacing U with Ψ.

^b^Predicted based on (39).

^c^Predicted based on (24).

^d^Predicted based on (19).

^e^Predicted based on (43).

^f^Predicted based on (36).

^g^Predicted based on (37).

^h^Predicted based on (40).

**Table 3.** Thermodynamic effect from replacing U with Ψ at middle position^a^

Sequences of the duplexes	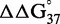 ^b^ (kcal/mol)	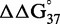 ^b^ (kcal/mol)	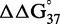 ^b^ (kcal/mol)	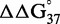 ^b^ (kcal/mol)
	X = A	X = G	X = U	X = C
5′ UCAG**Ψ**CAGU 3′	–0.71 ± 0.55 (–1.2)	–1.40 ± 0.34	–0.77 ± 0.14	–0.27 ± 0.05
3′ AGUC**X**GUCA 5′				
5′ UCAC**Ψ**GAGU 3′	–2.43 ± 0.49 (–0.8)	–0.43 ± 0.14^c^		
3′ AGUG**X**CUCA 5′				
5′ UCAA**Ψ**UAGU 3′	–0.27 ± 0.27 (–3.5)	–0.82 ± 0.10		
3′ AGUU**X**AUCA 5′				
5′ UCAU**Ψ**AAGU 3′	–0.55 ± 0.19 (–1.5)	–0.84 ± 0.07		
3′ AGUA**X**UUCA 5′				

^a^Solutions are 1 M NaCl, 20 mM sodium cacodylate and 0.5 mM Na_2_EDTA, pH 7.

^b^Differences in measured free energies relative to analogous RNA duplexes with uridine (U) instead of pseudouridine (Ψ); values in parenthesis are differences predicted on basis of nearest parameters (38) and (19); thermodynamic values used are from Tm^–1^ plots.

^c^Two transitions were observed when central pair was U-G, so two-state approximation is not valid.

### Contributions of terminal unpaired and paired Ψ on thermodynamic stabilities of RNA duplexes

Table 2 summarizes thermodynamic effects of adding terminal nucleotides U or Ψ as a 5′-dangling end on the C of a CG pair. U and Ψ enhanced free energy of duplex formation (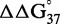) by similar increments of 0.78 and 0.66 kcal/mol, respectively, at 37°C. Both stability increments are more favorable than the 0.1 kcal/mol measured for the 5′-dangling U of (UCCGGp)_2_, where p represents a 3′-phosphate (39). Either U or Ψ as a 3′-dangling end on the G of a GC pair favors duplex (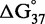) by 0.97 kcal/mol, within experimental error of the 0.6 kcal/mol measured for CCGGUp (40). It is commonly postulated that enhancement of RNA duplex stability by single 5′- and 3′-dangling ends are due to the stacking ability of the unpaired nucleotide (40–42). Based on NMR structural studies it is postulated that Ψ stacks better than U, presumably due to better rotational lability of the C1′-C5 glycoside bond in Ψ over the C1′-N1 in U (3). However, those conclusions were formulated for Ψ in an internal position of helical RNA.

Larger and sequence dependent stability increments are measured when a 5′ or 3′ terminal U or Ψ is paired with A, G, U or C. A 5′-terminal U-A in a 5′UC/3′AG nearest-neighbor sequence enhances duplex stability by 1.88 kcal/mol at 37°C, identical within error limits to the 1.81 kcal/mol measured for the 5′-terminal 5′ΨC/3′AG sequence (Table 1). A similar negligible difference is seen when U is replaced by Ψ in the 3′-terminal 5′GU/3′CA sequence, where addition of the terminal U-A or Ψ-A pairs provide stability increments of –2.18 and –2.10 kcal/mol, respectively. The increments of –1.88 and –2.18 kcal/mol for U are similar to increments of (–2.35 + 0.45) = –1.90 and (–2.24 + 0.45) = –1.79 kcal/mol expected from a nearest-neighbor model involving only Watson–Crick pairs (24). The increment of –1.81 kcal/mol for the terminal Ψ-A pair is similar to the value (–2.49 + 0.31) = –2.18 kcal/mol expected from a nearest-neighbor model, but the measured –2.10 kcal/mol is smaller than the predicted (–3.29 + 0.31) = –2.98 kcal/mol (19).

Adding a U-G and Ψ-G pair at the 5′ end enhanced stability by 1.78 (predicted is 1.80 kcal/mol, (43)) and 1.53 kcal/mol, respectively, which is within experimental error of the values for U-A and Ψ-A (Table 2). Placing U-G and Ψ-G pairs at the 3′ end enhanced stabilities by 2.44 (predicted is 2.15 kcal/mol) and 3.22 kcal/mol, respectively, suggesting some extra stabilization from the 3′-terminal Ψ-G. Adding a U-U or Ψ-U pair at the 5′ end enhanced stability by 0.86 and 1.41 kcal/mol, respectively, similar to values of 1.05 and 1.83 kcal/mol for the additions at the 3′ end. Here Ψ-U appears more stable than U-U at both ends. Adding a U-C or Ψ-C pair at the 5′ end enhanced stability by 1.12 and 0.81 kcal/mol, respectively, which are close, considering experimental errors. The values at the 3′ end are 1.36 and 1.52 kcal/mol, respectively, also within experimental error.

The results summarized in Table 2 reveal that in most cases the thermodynamic effect of substitution of Ψ for U at terminal positions is within experimental error. The exceptions are 3′ terminal U-G and U-U pairs in the 5′GU/3′CG and 5′GU/3′CU nearest-neighbor motifs, respectively, where replacement of U with Ψ enhances stability by 0.8 kcal/mol. In all but two cases, the terminal stability increments are also consistent with previous measurements. One difference is that the 5′ dangling end U in the 5′UC/3′G motif had a stability increment of –0.78 kcal/mol compared to a previous single measurement of –0.1 kcal/mol. It is possible that the enhanced stability reflects a dynamic pairing ensemble of the 3′G with 5′UC relative to that possible with the previous measurement on the shorter duplex (UCCGGp)_2_. Another possibility is an unexpected effect from the 3′ phosphates in (UCCGGp)_2._ The other case where the measurement is not predicted within experimental error is the 3′ terminal 5′GΨ/3′CA motif where the measured stability increment is –2.10 kcal/mol compared to a nearest-neighbor prediction of –2.98 kcal/mol. The nearest-neighbor parameters for Ψ-A pairs, however, are currently based on only five occurrences of each (19).

### Contribution of internal paired Ψ on thermodynamic stabilities of RNA duplexes

Somewhat larger effects were measured when U was replaced by Ψ in Ψ-A, Ψ-G, Ψ-U and Ψ-C pairs at internal positions (Table 3). In most cases, replacing U with Ψ clearly enhanced thermodynamic stabilities (i.e. gave more negative 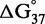).

Most enhancements in stabilities upon replacing U with Ψ to give internal Ψ-A, Ψ-G and Ψ-U pairs ranged from 0.3 to 0.8 kcal/mol (Table 3). The two outliers are the motifs 5′CΨG/3′GAC and 5′GΨC/3′CGG where Ψ substitution for U enhances duplex stability by 2.43 and 1.40 kcal/mol, respectively. In these cases, flipping the Watson–Crick base pair orientation gives enhancements of 0.71 and ∼0.4 kcal/mol, respectively, which is within the usual range of 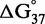
’s. Presumably, the outliers reflect favorable stacking and hydrogen bonding interactions of Ψ relative to U. As shown below, there is no NMR evidence for hydrogen bonding involving the H1 imino proton of Ψ in the 5′GΨC/3′CGG duplex. The results suggest that 3D structures and computational studies of the outlier duplexes might be interesting because little is known about interactions favoring stacking.

Because nearest-neighbor parameters are available for U-A and Ψ-A pairs adjacent to Watson–Crick pairs, it is possible to compare the measured and predicted effects of replacing a middle U-A pair with Ψ-A (Table 3). In two cases, the difference between measured and predicted exceeds 1 kcal/mol. The measured versus predicted values are –2.43 versus –0.8 kcal/mol for the 5′CΨG/3′GAC central motif and –0.27 versus –3.5 kcal/mol for the 5′AΨU/3′UAA central motif. The 5′AΨU/3′UAA motif occurs in the duplex predicted least well by nearest-neighbor parameters (Table 1). It also contains the two Ψ-A nearest-neighbor parameters (19) that differ most from their U-A (24) equivalents with Ψ enhancements of –1.70 and –1.81 kcal/mol.

### NMR reveals at least one hydrogen bond of pseudouridine pairing with A, G or U

**Figure 2.**
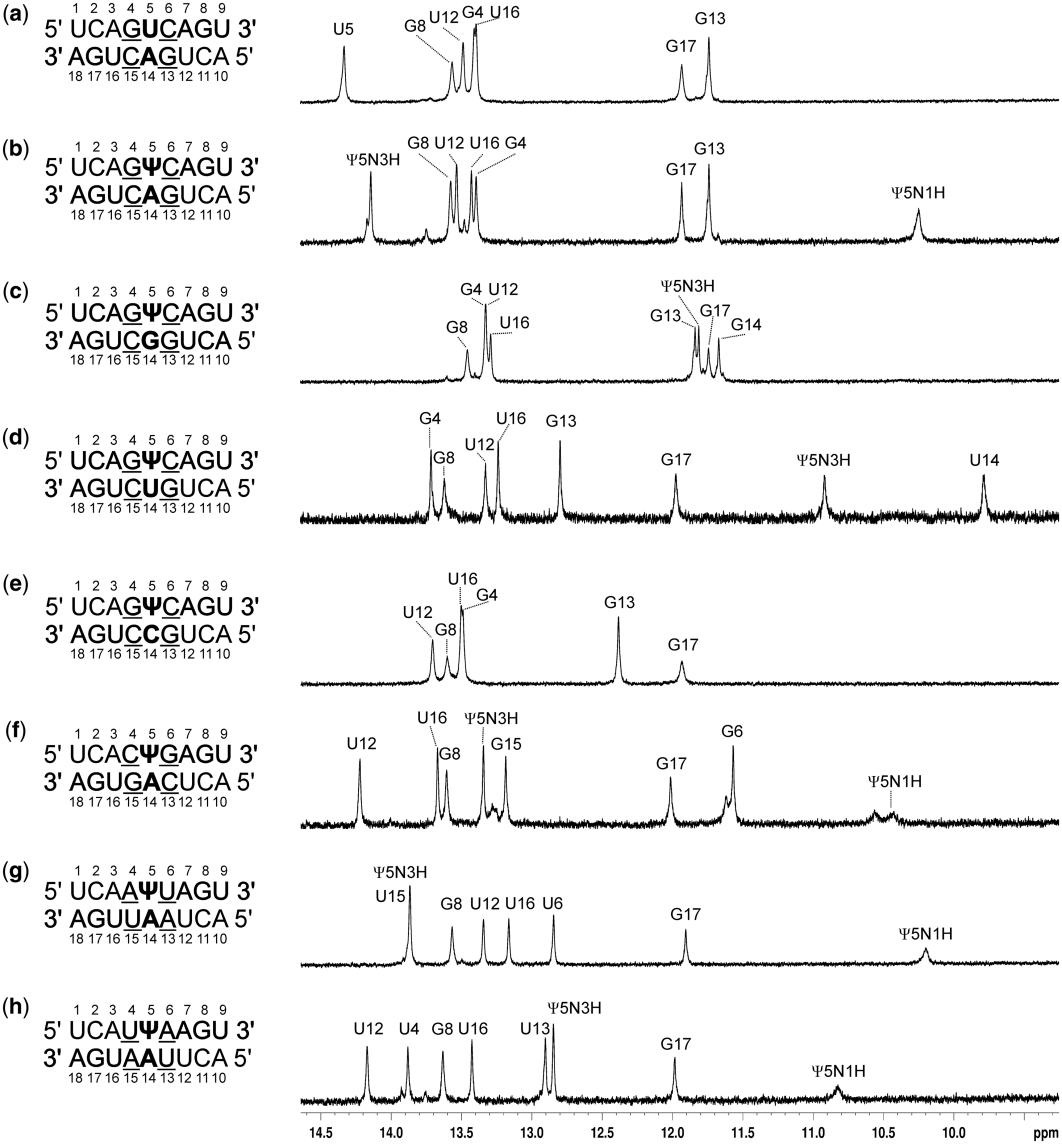
1D NMR spectra. Buffer is 150 mM NaCl, 10 mM sodium phosphate and 0.1 mM EDTA, pH 6.8. For spectra (**a**), (**b**) and (**c**) to (**h**), duplex concentrations were 1.5, 1.0 and 0.5 mM, respectively.

Pseudouridine is potentially more versatile in its hydrogen bonding than uridine because two imino protons are available to serve as hydrogen bond donors (Figure 1). NMR spectroscopy was used to compare hydrogen bonding in a central U-A pair with that with a central Ψ paired with A, C, G or U (Figure 2).

#### NMR of duplexes with a U-A or Ψ-A central pair

The 1D proton spectrum of the duplex containing a U_5_-A_14_ pair revealed seven imino proton resonances. The G and U imino protons were distinguished based on analysis of ^15^N chemical shifts in ^1^H-^15^N HSQC spectra and by characteristic NOE cross-peaks to C amino protons for G and to AH2 protons for U. Assignments were made with 2D NOESY spectra (data not shown), which revealed typical intra- and inter-strand correlations. No imino resonances were detected for the terminal U’s. Non-exchangeable protons were assigned with standard methods (44). The NOESY spectra recorded in D_2_O exhibited typical features of double-stranded sequential connectivities and are consistent with a duplex structure in which all nucleotides are involved in Watson–Crick base pairs.

The 1D proton spectrum of the Ψ-A duplex (Figure 2) shows eight major imino resonances and is similar to that of the U-A duplex except for the appearance of a new signal at 10.25 ppm. This high field resonance was assigned to the Ψ_5_N1H imino proton and its chemical shift is in agreement with previous observations of Ψ-A base pairs (8,17,45). Assignments of Ψ_5_N1H resonances throughout this work were based on the fact that they exhibit strong NOEs to upfield peaks assigned as Ψ_5_CH6. The Ψ_5_N3H imino proton is shifted upfield by ∼0.19 ppm relative to the U_5_N1H imino proton of the U-A duplex. The presence of NOE cross-peaks between Ψ_5_N3H and G_4_N1H and G_13_N1H was helpful in their unambiguous assignments. The observed strong NOE cross correlation between Ψ_5_N3H and the A_14_H2 proton (data not shown) demonstrated that pseudouridine Ψ_5_ pairs with adenosine A_14_ in a Watson–Crick manner involving Ψ_5_N3H-A_14_N1 and Ψ_5_O2-A_14_N6H hydrogen bonds (Figure 1).

Spectra were similarly measured, assigned and interpreted for duplexes with a central Ψ-A in triplets 5′CΨG/3′GAC, 5′AΨU/3′UAA and 5′UΨA/3′AAU (Figure 2). In all cases, the Ψ-A pair is in a Watson–Crick conformation. The spectra for the 5′AΨU/3′UAA, and 5′UΨA/3′AAU duplexes show eight resonances (this includes an overlap of U_15_N3H and Ψ_5_N3H for 5′AΨU/3′UAA), with the Ψ_5_N1H resonance upfield between 10 and 11 ppm. The spectrum of the 5′CΨG/3′GAC duplex, however, has two resonances between 10 and 11 ppm suggesting some conformational equilibrium involving Ψ_5_N1H. The appearance and upfield chemical shift of the Ψ_5_N1H is consistent with those protons being partially protected from exchange with water and potentially in a weak hydrogen bond.

#### NMR of duplexes with a Ψ-G, Ψ-U or Ψ-C central pair

The 1D proton spectrum of the Ψ-G duplex (Figure 2) shows eight imino proton resonances because the resonances of G_4_ and U_12_ are overlapped at 13.50 ppm. A strong imino–imino cross-peak was observed between resonances at 11.99 and 11.85 ppm and the chemical shifts were attributed to Ψ_5_N3H and G_14_N1H, respectively. This type of interaction is distinctive of wobble pair formation because of the short distance between the two imino protons (Figure 1).

The 1D proton spectrum (Figure 2) of the Ψ-U duplex shows eight imino proton resonances. The Ψ_5_N3H imino proton was distinguished from U_14_N3H by observation of an NOE cross-peak between the amino proton of C_6_ and Ψ_5_N3H (data not shown). A strong NOE cross-peak between Ψ_5_N3H and U_14_N3H protons was observed indicating formation of a Ψ-U base pair. Based on NOESY data alone, however, it was not possible to distinguish between the types of Ψ-U base pairs shown in Figure 1.

The proton spectrum of the Ψ-C duplex (Figure 2) shows only six imino proton resonances and all were assigned to Watson–Crick A-U and G-C pairs. No imino protons of Ψ were detected, presumably due to exchange with water. Evidently, Ψ and C do not hydrogen bond together. Nevertheless, a continuous set of H8/H6-H1′ NOE connectivities including Ψ_5_ and C_14_, were determined for both strands, so both residues stack into the duplex. Additionally, the presence of aromatic–aromatic NOE cross-peaks between G_4_H8/Ψ_5_H6, Ψ_5_H6/C_6_H6, G_13_H8/C_14_H6 and C_14_H6/C_15_H6 support inward orientation of Ψ_5_ and C_14_. The lack of hydrogen bonding in the Ψ-C pair could explain why the 5′GΨC/3′CCG duplex is the least stable of those with a central 5′GΨC/3′CNG motif.

### CD spectra of RNA duplexes containing pseudouridine

**Figure 3.**
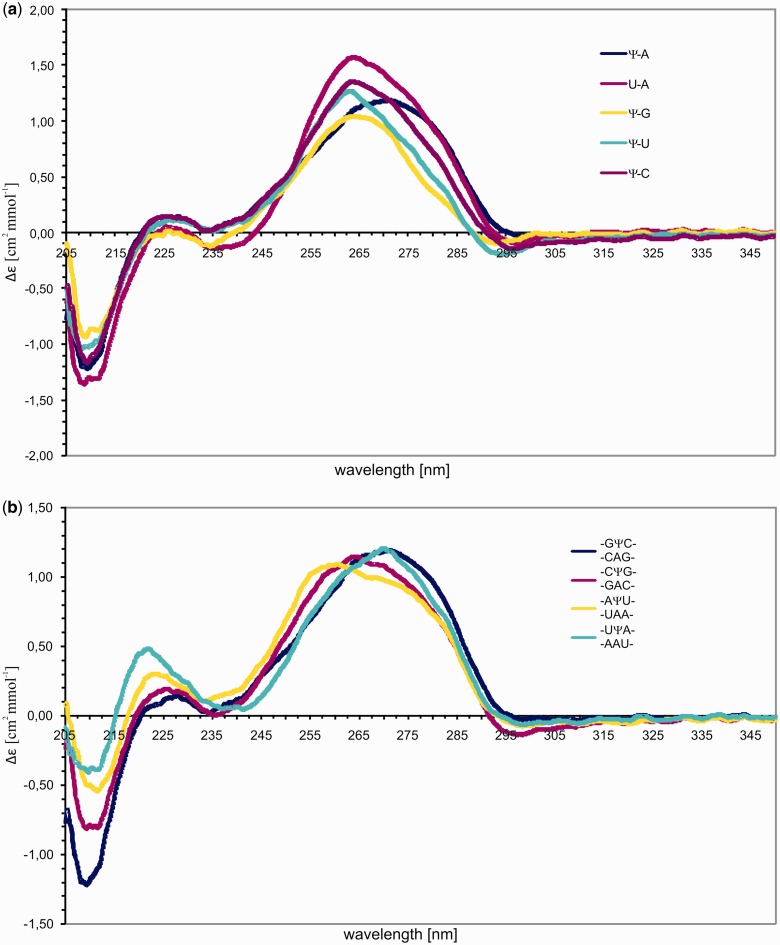
CD spectra of RNA duplexes. (**a**) Spectra of duplexes 5′UCAGMCAGU/3′AGUCNGUCA containing at central position U-A, Ψ-A, Ψ-G, Ψ-U and Ψ-C, (**b**) Spectra of duplexes containing at central position Ψ-A surrounded by various 5′- and 3′-adjacent base pairs.

The influence of Ψ residues on global helical structure was investigated by measuring CD spectra for all the duplexes listed in Table 1. Spectra at 10°C for some of the duplexes with internal Ψ are shown in Figure 3 (see also Supporting Information). The spectra have shapes consistent with A-form RNA helixes (46,47), although some shifting and changing of intensity of peaks, shifting of crossover points and appearance of some spectral shoulders were observed, consistent with literature reports (3,5,7,8,17). CD spectra of duplexes with U instead of Ψ exhibited similar variety. Evidently, internal substitution of Ψ for U has little effect on global conformation.

Surprisingly, substitution of Ψ for U as a 5′-dangling end diminished by ca. half the molar ellipticity of the positive peak at 266 nm and reduced even more the intensity of the negative peak at 209 nm (Supporting Information). The CD spectra with and without a 5′-dangling U were essentially identical. When Ψ or U was placed as a 3′- dangling end, the CD spectra were nearly identical. Less intense CD spectra were also observed when Ψ replaced the 5′U in U-A, U-G and U-U pairs. All the spectra with 5′-terminal Ψ had ellipticity of less than or ∼1 near 265 nm. Most other spectra had ellipticities between 1 and 2 in this region. No duplexes with ellipticity >2 near 265 nm had internal or 5′ terminal Ψ. The CD spectra did not reveal any structural reasons for the thermodynamic results.

## DISCUSSION

It has been shown that replacement of U with Ψ thermodynamically stabilizes RNA duplexes (3,8,9,18). Usually stronger stacking of Ψ in comparison to U was postulated as the major origin of enhancement of stability (3). Recently, Znosko’s team quantified the effect for Ψ-A pairs relative to U-A pairs by determination of nearest-neighbor parameters for Ψ-A on the basis of optical melting of 24 duplexes (19). They found that on average at internal and terminal positions, substitution of Ψ-A for U-A enhanced duplex stability by 1.7 and 1.0 kcal/mol, respectively. As shown in Table 1, the nearest-neighbor parameters for Ψ-A and for Watson–Crick pairs predict within 1 kcal/mol the measured 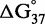
’s for five of the six duplexes with Ψ-A pairs, when experimental error is considered. The exception has a central motif of 5′AΨU/3′UAA and is predicted to be more stable than measured by 3 kcal/mol at 37°C. This duplex contains the two Ψ-A nearest neighbors with parameters that differ most from those of U-A, suggesting an unusual context dependence for stabilities of 5′AΨ/3′UA and 5′ΨA/3′AU nearest neighbors.

Tables 1–3 present for the first time thermodynamic effects of substituting Ψ for U to give Ψ-G, Ψ-U and Ψ-C pairs. Such pairs are often found in pseudouridylated RNA duplexes, particularly of splicesomal snRNAs (13). Considering experimental error, substituting Ψ for U in these mispairs can enhance stability between ∼0 and 1 kcal/mol, with an average of 0.5 kcal/mol (Tables 2 and 3). The largest stabilization after Ψ-A is for Ψ-G, whereas Ψ-C has little effect. For the six duplexes with Ψ-G, enhancements of stabilities relative to U-G duplexes range between 0 and 1.4 kcal/mol (Tables 2 and 3). These initial results imply that nearest-neighbor parameters for Ψ-G pairs will be dependent on the neighbor, as already known for Watson–Crick (24), Ψ-A (19) and U-G (43) pairs.

NMR studies of duplexes with internal Ψ-A, Ψ-G and Ψ-U pairs revealed hydrogen bonding via the N3 imino proton. The spectra are consistent with Ψ and U hydrogen bonding to A, G and U in similar ways (Figure 2). Resonances are also seen for ΨN1H proton in duplexes with a Ψ-A pair, suggesting ΨN1H may be involved in a weak hydrogen bond. In contrast, no imino proton resonances were observed for Ψ-C, implying a lack of hydrogen bonding. This correlates with Ψ-C duplexes being the least stable. CD spectra are consistent with expectations from NMR spectra in that the overall global conformation is A-form with or without Ψ.

## SUPPLEMENTARY DATA

Supplementary Data are available at NAR Online.

## FUNDING

National Science Center [UMO-2011/03/B/NZ1/00576, UMO-2011/03/B/ST5/01098, UMO-2013/08/A/ST5/00295 to R.K.; N N301 788 440, UMO-2013/08/M/NZ1/01062 to E.K.; N N301 707040 to Z.G.]; National Institutes of Health (NIH) [1R03TW008739-01 to E.K. and D.H.T.; GM22939 to D.H.T.]; Ministry of Science and Higher Education and European Fund for Regional Development [UDA-POIG.02.01.00-30-182/09]. Funding for open access charge: Corresponding author is Nucleic Acids Research Editorial Board member.

*Conflict of interest statement*. None declared.

## Supplementary Material

Supplementary Data
