# Supplementary material for: The contribution of pseudouridine to stabilities and structure of RNAs
Source: Nucleic Acids Res. 2013 Dec 24;42(5):3492–501. doi: 10.1093/nar/gkt1330 (PMC3950712; doi:10.1093/nar/gkt1330)
Supplement: Supplementary Data [file supp_42_5_3492__index.html]

The contribution of pseudouridine to stabilities and structure of RNAs — Supplementary Data 

# The contribution of pseudouridine to stabilities and structure of RNAs

## Supplementary Data

files

**Files in this Data Supplement:**

- Supplementary Data - doc file
