# Supplementary material for: The contribution of pseudouridine to stabilities and structure of RNAs
Source: Nucleic Acids Res. 2013 Dec 24;42(5):3492–501. doi: 10.1093/nar/gkt1330 (PMC3950712; doi:10.1093/nar/gkt1330)
Supplement: Supplementary Data [file supp_gkt1330_nar-03056-r-2013-File002.doc]

**SUPPLEMENTARY DATA**

**THE CONTRIBUTION OF PSEUDOURIDINE TO STABILITIES AND STRUCTURE OF RNAs.**

Elzbieta Kierzek, Magdalena Malgowska, Jolanta Lisowiec, Douglas H. Turner^#^, Zofia Gdaniec and Ryszard Kierzek*

Institute of Bioorganic Chemistry Polish Academy of Sciences, 61-704 Poznan, Noskowskiego 12/14, Poland

^#^ Department of Chemistry, University of Rochester, Rochester, New York 14627, United States

* corresponding author, (Phone) +4861-853-8503, (Fax) +4861-852-0532, E-mail: [rkierzek@ibch.poznan.pl](mailto:rkierzek@ibch.poznan.pl)


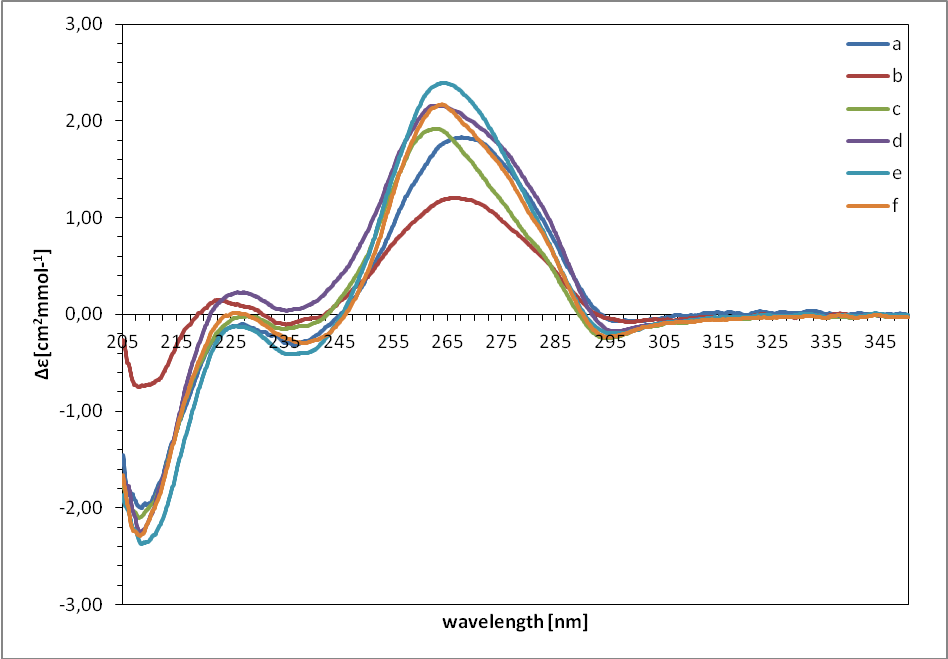


**Figure S1**. CD spectra of RNA duplexes. (a) 5’**U**CAGUCAGU/3’GUCAGUCA, (b) 5’**Ψ**CAGUCAGU/3’GUCAGUCA, (c) 5’UCAGUCAG**U**/3’AGUCAGUC, (d) 5’UCAGUCAG**Ψ**/3’AGUCAGUC, (e) 5’CAGUCAGU/3’GUCAGUCA (f) 5’UCAGUCAG/3’AGUCAGUC.


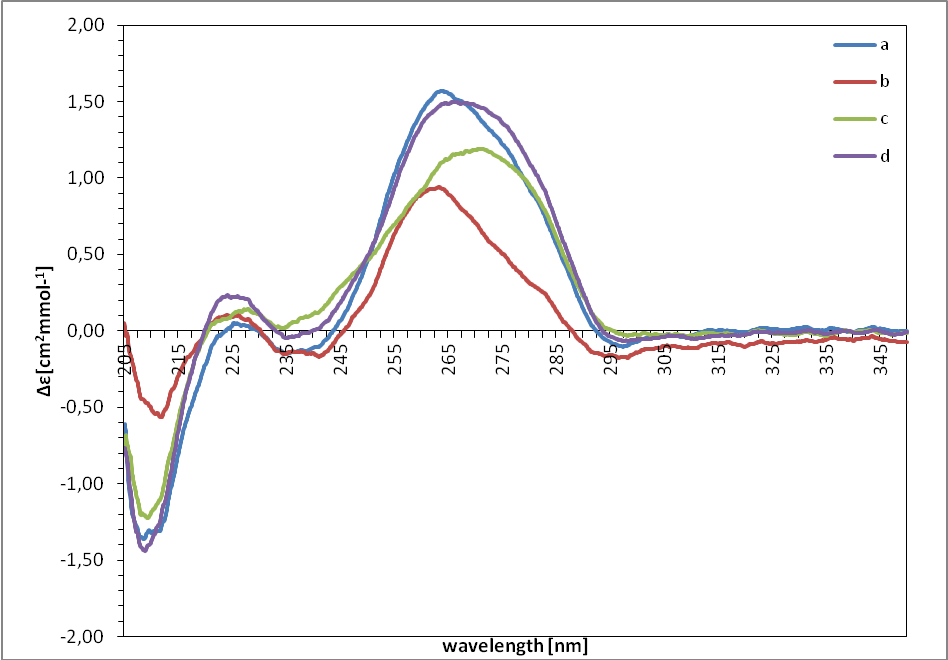


**Figure S2.** CD spectra of RNA duplexes. (a) 5’**U**CAGUCAGU/3’**A**GUCAGUCA, (b) 5’**Ψ**CAGUCAGU/3’**A**GUCAGUCA, (c) 5’UCAGUCAG**U**/3’ AGUCAGUC**A,** (d) 5’UCAGUCAG**Ψ**/3’AGUCAGUC**A.**


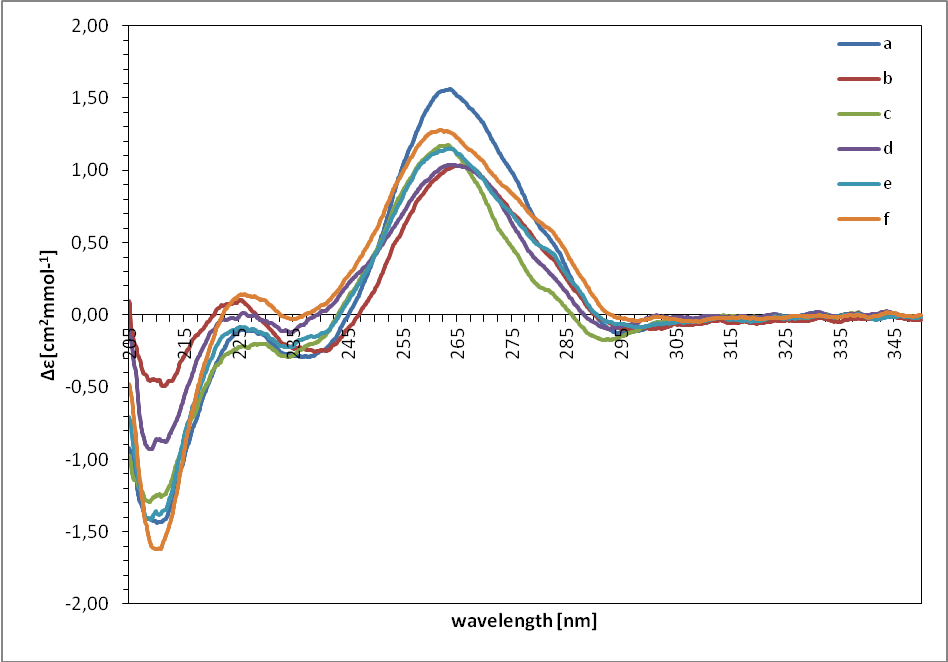


**Figure S3.** CD spectra of RNA duplexes. (a) 5’**U**CAGUCAGU/3’**G**GUCAGUCA, (b) 5’**Ψ**CAGUCAGU/3’**G**GUCAGUCA, (c) 5’UCAG**U**CAGU/3’AGUC**G**GUCA, (d) 5’UCAG**Ψ**CAGU/3’AGUC**G**GUCA, (e) 5’UCAGUCAG**U**/3’AGUCAGUC**G** (f) 5’UCAGUCAG**Ψ**/3’AGUCAGUC**G.**


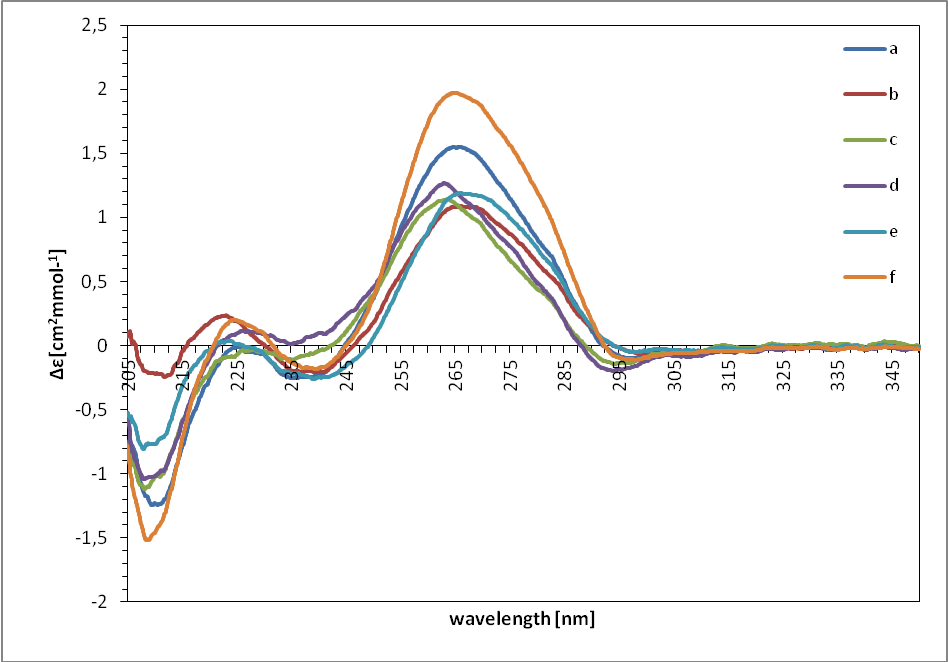


**Figure S4**. CD spectra of RNA duplexes. (a) 5’**U**CAGUCAGU/3’**U**GUCAGUCA, (b) 5’**Ψ**CAGUCAGU/3’**U**GUCAGUCA, (c) 5’UCAG**U**CAGU/3’AGUC**U**GUCA, (d) 5’UCAG**Ψ**CAGU/3’AGUC**U**GUCA, (e) 5’UCAGUCAG**U**/3’AGUCAGUC**U,** (f) 5’UCAGUCAG**Ψ**/3’AGUCAGUC**U.**


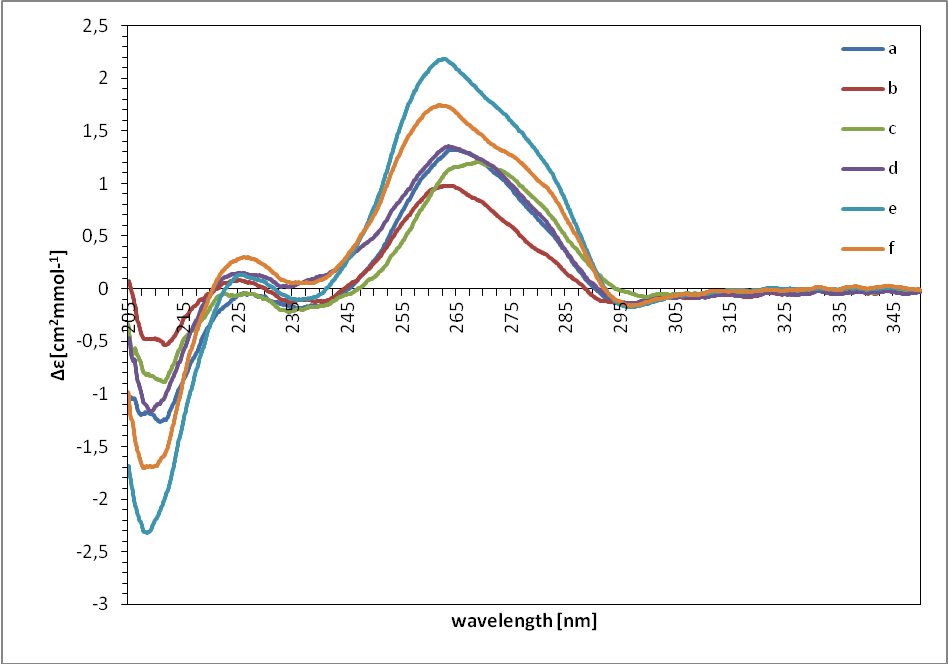


**Figure S5.** CD spectra of RNA duplexes. (a) 5’**U**CAGUCAGU/3’**C**GUCAGUCA, (b) 5’**Ψ**CAGUCAGU/3’**C**GUCAGUCA, (c) 5’UCAG**U**CAGU/3’AGUC**C**GUCA, (d) 5’UCAG**Ψ**CAGU/3’AGUC**C**GUCA, (e) 5’UCAGUCAG**U**/3’AGUCAGUC**C,** (f) 5’UCAGUCAG**Ψ**/3’AGUCAGUC**C.**


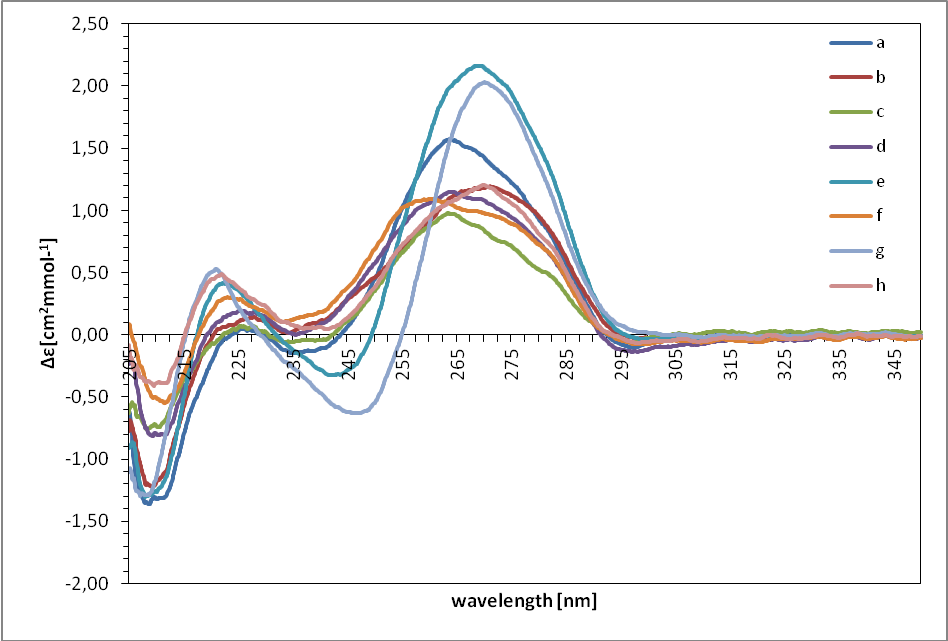


**Figure S6.** CD spectra of RNA duplexes. (a) 5’UCAG**U**CAGU/3’AGUC**A**GUCA, (b) 5’UCAG**Ψ**CAGU/3’AGUC**A**GUCA, (c) 5’UCAC**U**GAGU/3’AGUG**A**CUCA, (d) 5’UCAC**Ψ**GAGU/3’AGUG**A**CUCA, (e) 5’UCAA**U**UAGU/3’AGUU**A**AUCA, (f) 5’UCAA**Ψ**UAGU/3’AGUU**A**AUCA, (g) 5’UCAU**U**AAGU/3’AGUA**A**UUCA, (h) 5’UCAU**Ψ**AAGU/3’AGUA**A**UUCA.


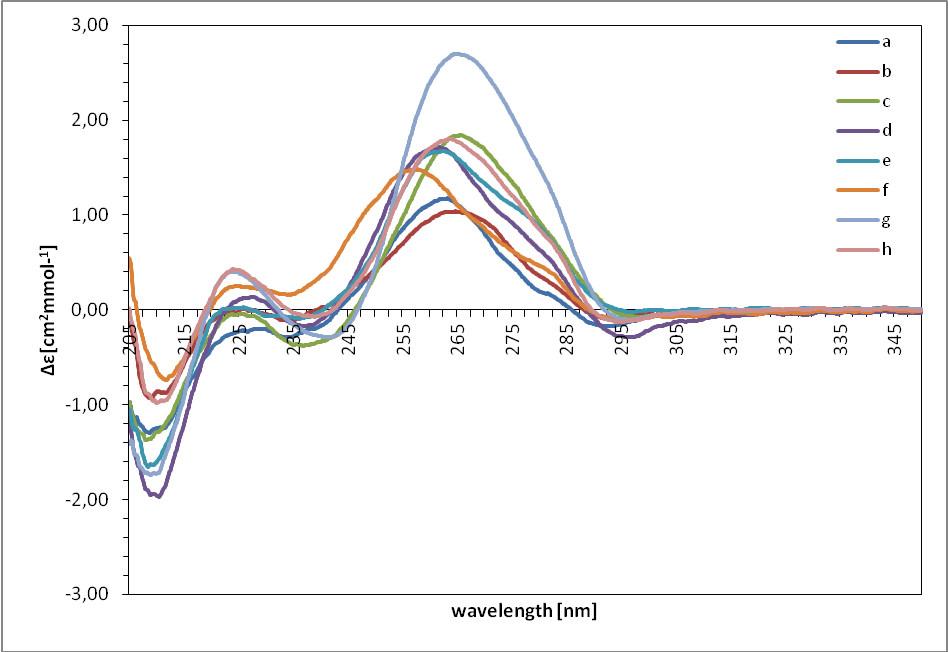


**Figure S7.** CD spectra of RNA duplexes. (a) 5’UCAG**U**CAGU/3’AGUC**G**GUCA, (b) 5’UCAG**Ψ**CAGU/3’AGUC**G**GUCA, (c) 5’UCAC**U**GAGU/3’AGUG**G**CUCA, (d) 5’UCAC**Ψ**GAGU/3’AGUG**G**CUCA, (e) 5’UCAA**U**UAGU/3’AGUU**G**AUCA, (f) 5’UCAA**Ψ**UAGU/3’AGUU**G**AUCA, (g) 5’UCAU**U**AAGU/3’AGUA**G**UUCA, (h) 5’UCAU**Ψ**AAGU/3’AGUA**G**UUCA.


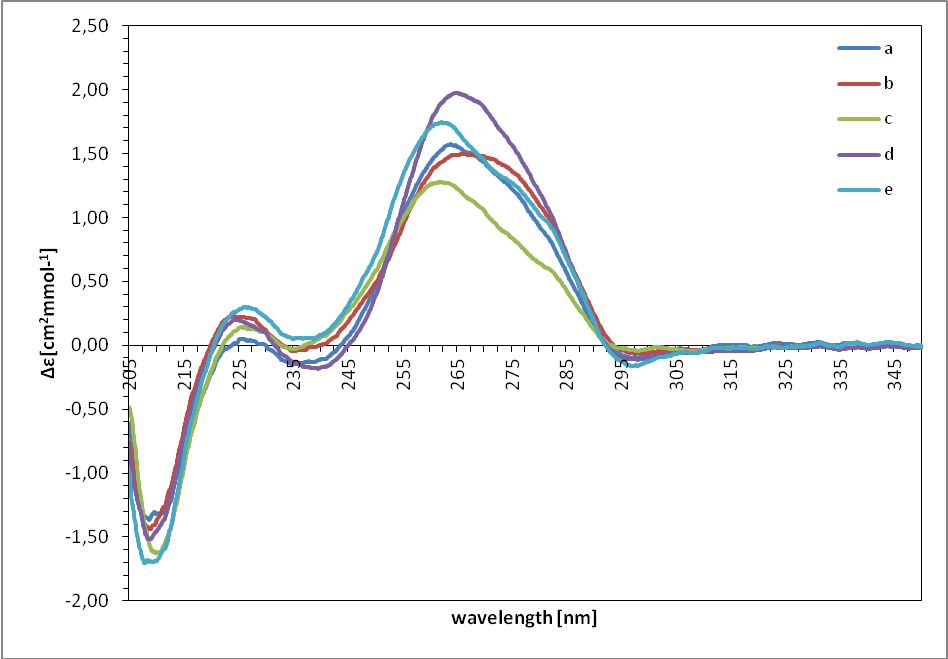


**Figure S8.** CD spectra of RNA duplexes. (a) 5’UCAGUCAG**U**/3’AGUCAGUC**A,** (b) 5’UCAGUCAG**Ψ**/3’AGUCAGUC**A,** (c) 5’UCAGUCAG**Ψ**/3’AGUCAGUC**G,** (d) 5’UCAGUCAG**Ψ**/3’AGUCAGUC**U,** (e) 5’UCAGUCAG**Ψ**/3’AGUCAGUC**C.**


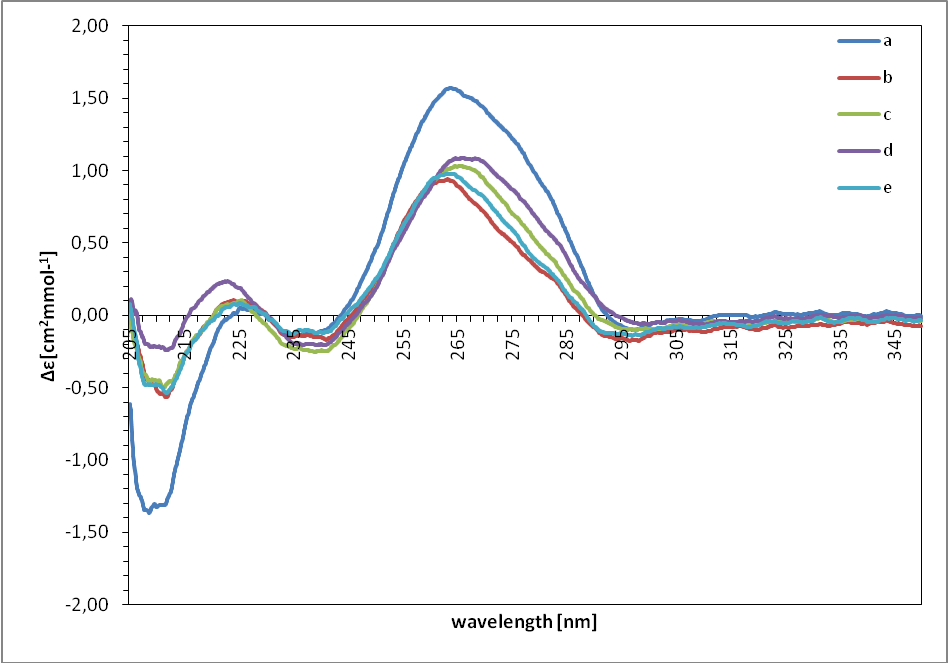


**Figure S9.** CD spectra of RNA duplexes. (a) 5’**U**CAGUCAGU/3’**A**GUCAGUCA, (b) 5’**Ψ**CAGUCAGU/3’**A**GUCAGUCA, (c) 5’**Ψ**CAGUCAGU/3’**G**GUCAGUCA, (d) 5’**Ψ**CAGUCAGU/3’**U**GUCAGUCA, (e) 5’**Ψ**CAGUCAGU/3’**C**GUCAGUCA.


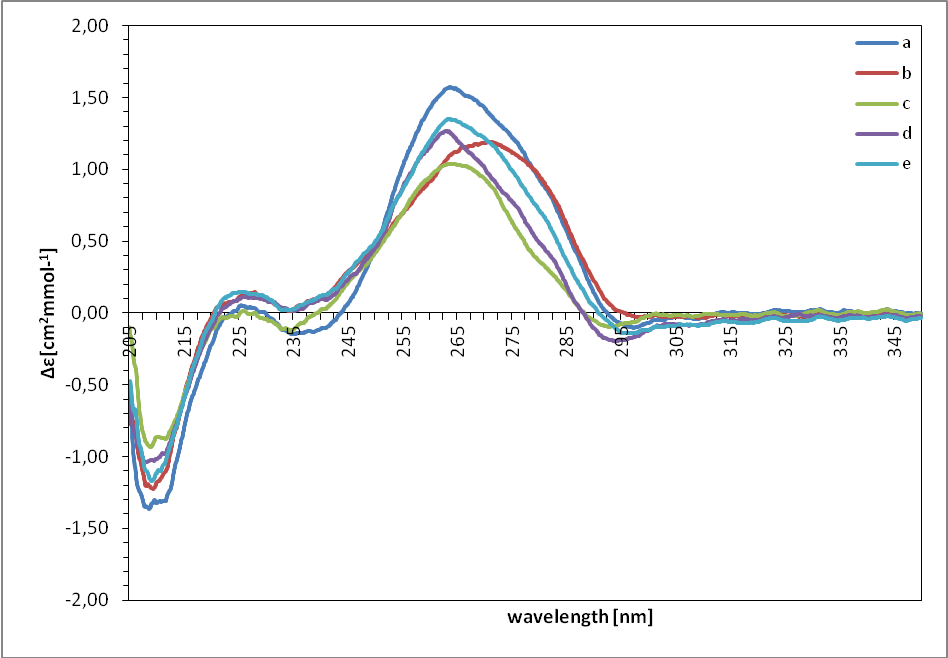


**Figure S10.** CD spectra of RNA duplexes. (a) 5’UCAG**U**CAGU/3’AGUC**A**GUCA, (b) 5’UCAG**Ψ**CAGU/3’AGUC**A**GUCA, (c) 5’UCAG**Ψ**CAGU/3’AGUC**G**GUCA, (d) 5’UCAG**Ψ**CAGU/3’AGUC**U**GUCA, (e) 5’UCAG**Ψ**CAGU/3’AGUC**C**GUCA.

**Figure S11**. Reciprocal melting temperature versus log concentration plots for 5’**Ψ**CAGUCAGU/3’**A**GUCAGUCA (P1/R1), 5’UCAG**Ψ**CAGU/3’AGUC**A**GUCA (P2/R1), 5’UCAC**Ψ**GAGU/3’AGUG**A**CUCA (P4/R17), 5’UCAA**Ψ**UAGU/3’AGUU**A**AUCA (P5/R18), 5’UCAU**Ψ**AAGU/3’AGUA**A**UUCA (P6/R19), 5’UCAGUCAG**Ψ**/3’AGUCAGUC**A** (P3/R1).
